# Relation of α_2_-Antiplasmin Genotype and Genetic Determinants of Fibrinogen Synthesis and Fibrin Clot Formation with Vascular Endothelial Growth Factor Level in Axial Spondyloarthritis

**DOI:** 10.3390/ijms21249383

**Published:** 2020-12-09

**Authors:** Berthold Hoppe, Christian Schwedler, Hildrun Haibel, Maryna Verba, Fabian Proft, Mikhail Protopopov, Hans-Gert Heuft, Valeria Rios Rodriguez, Anke Edelmann, Martin Rudwaleit, Joachim Sieper, Denis Poddubnyy

**Affiliations:** 1 Institute of Laboratory Medicine, Unfallkrankenhaus Berlin, 12683 Berlin, Germany; 2 Institute of Laboratory Medicine, Clinical Chemistry and Pathobiochemistry, Charité–Universitätsmedizin Berlin, Corporate Member of Freie Universität Berlin, Humboldt-Universität zu Berlin and Berlin Institute of Health, 10117 Berlin, Germany; Christian.schwedler@charite.de; 3 Department of Gastroenterology, Infectious Diseases and Rheumatology, Charité–Universitätsmedizin Berlin, 12200 Berlin, Germany; Hildrun.Haibel@charite.de (H.H.); Maryna.Verba@charite.de (M.V.); Fabian.Proft@charite.de (F.P.); Mikhail.Protopopov@charite.de (M.P.); Valeria.Rios-Rodriguez@charite.de (V.R.R.); Martin.Rudwaleit@klinikumbielefeld.de (M.R.); Joachim.Sieper@charite.de (J.S.); Denis.Poddubnyy@charite.de (D.P.); 4 Institute of Transfusion Medicine, Otto-von-Guericke Universität Magdeburg, 39120 Magdeburg, Germany; Hans-Gert.Heuft@med.ovgu.de; 5 Department of Molecular Diagnostics, Labor Berlin–Charité Vivantes GmbH, 13353 Berlin, Germany; Anke.Edelmann@laborberlin.com; 6 Department of Internal Medicine and Rheumatology, Klinikum Bielefeld Rosenhöhe, 33647 Bielefeld, Germany; 7 Department of Epidemiology, German Rheumatism Research Centre, 10117 Berlin, Germany

**Keywords:** fibrinolysis, α_2_-antiplasmin, fibrinogen, factor XIII, spondyloarthritis, vascular endothelial growth factor

## Abstract

Objective: Coagulation and fibrinolysis are interrelated with the expression of vascular endothelial growth factor (VEGF), which frequently is increased in axial spondyloarthritis (axSpA). We tested whether (i) α_2_-antiplasmin (A2AP) Arg6Trp, (ii) fibrinogen, factor XIII A-subunit or B-subunit genotypes are associated with VEGF levels and assessed whether the known association between elevated VEGF and radiographic spinal progression in axSpA depends on genetic background. Methods: One hundred and eighty-six axSpA patients from the German Spondyloarthritis Inception Cohort were genotyped, characterized for VEGF levels, and statistically analyzed. The association between VEGF and radiographic spinal progression was assessed in dependence on genetic background in stratified analyses. Results: A2AP 6Trp carriage was associated with VEGF elevation (OR: 2.37, 95% CI: 1.06–5.29) and VEGF levels (6Trp, 455 ± 334 pg/mL; 6Arg/Arg, 373 ± 293 pg/mL; *p* < 0.008). Association between elevated VEGF and radiographic spinal progression in axSpA (OR: 3.11, 95% CI: 1.02–8.82) depended remarkably on the fibrinogen (FGA) genotype. When considering axSpA patients with elevated VEGF, in FGA rs6050A>G wild types, 42.1% of patients (8 of 19) progressed, while in G-allele carriers, no radiographic progression happened (0 of 13) (*p* < 0.04). Conclusions: The A2AP Arg6Trp genotype seems to influence VEGF levels in axSpA. The predictive value of VEGF elevations in respect of radiographic spinal progression in axSpA depends on FGA genotypes.

## 1. Introduction

There is a complex interrelation between coagulation, fibrinolysis, inflammation, and wound healing [1,2,3,4,5]. Following an injury, coagulation assures spatial localization and the stabilization of endothelial cells [1]. Afterwards, proteins of the coagulation system as well as peptide fragments generated during coagulation and fibrinolysis allow for transendothelial leukocyte migration [6,7], and they also support and modulate angiogenesis necessary for wound healing [1]. In this context, vascular endothelial growth factor (VEGF) plays an important role as a decisive regulator of vascular permeability, angiogenesis, inflammation, and tissue repair [8]. The expression of VEGF is dependent on plasminogen and thrombomodulin signaling [9]. α_2_-Antiplasmin (A2AP) seems to be involved in the regulation of VEGF expression, as well [2,3], and it also has been described to modulate VEGF-dependent signaling pathways [10]. After secretion, VEGF is sequestrated in the fibrin gel matrix, which is presumably mediated by indirectly binding to fibrin via heparin [11]. The fibrin clot acts as a reservoir continuously releasing VEGF [11], and the binding of VEGF to fibrin enhances its capacity to induce endothelial proliferation [12]. Consequently, VEGF directly or indirectly triggers a cascade, which results in the recruitment of inflammatory cells, in increased vascular permeability and cytokine release, and in endothelial activation and revascularization [11].

In axial spondyloarthritis (axSpA), which is a chronic inflammatory disease of high frequency and which affects the axial skeleton [13], currently available data suggest that initial inflammation in the vertebral bodies is followed by an activation of repair processes with the formation of fibrous tissue, activation of osteoblasts, and finally the formation of so-called syndesmophytes, i.e., heterotopic ossification of spinal ligaments or of the annulus fibrosus [14]. By influencing inflammation and angiogenesis, VEGF is strongly involved in bone repair and regeneration [11], and it has been described to be a prognostic biomarker for axSpA, with elevated VEGF levels being associated with an increased risk for radiographic progress of the disease [15].

A2AP is the main inhibitor of fibrinolysis when incorporated into the fibrin clot. The A2AP Arg6Trp (rs2070863) genotype is known to diminish A2AP processing, hereby reducing A2AP incorporation, which results in A2AP-poor, fibrinolysis-susceptible fibrin nets [16]. Furthermore, the genetics of A2AP, fibrinogen, and factor XIII are known to influence fibrinogen synthesis [17,18], fibrin clot formation, and/or fibrinolysis resistance [19,20,21] as well as inflammatory activity [5,22,23].

Based on the presumed importance of VEGF in the pathogenesis of axSpA and on the data described above, which indicate a relation between fibrin clot formation and fibrinolysis with VEGF expression, we tested the following hypotheses in a cohort of axSpA patients. (i) The A2AP Arg6Trp (rs2070863) genotype is associated with increased VEGF levels in axSpA. (ii) Those genotype constellations of fibrinogen and factor XIII, which are known or assumed to be related to lower fibrin clot densities, i.e., reduced crosslinking and higher susceptibility to fibrinolysis, are associated with increased VEGF levels. Finally, (iii) we exploratorily tested whether the known association between elevated VEGF levels and radiographic spinal progression in axSpA [15] would depend on genotypes related to fibrinogen synthesis, fibrin clot formation, or fibrinolysis.

## 2. Results

### 2.1. Clinical Characteristics and Genotype Distribution

A total of 186 out of 210 patients of German Spondyloarthritis Inception Cohort (GESPIC) with clinical and radiographic follow-up after two years could be included in this study. The baseline characteristics of these patients are presented in Table 1.

Compared to axSpA patients without significant heterotopic ossification in X-rays (nr-axSpA), those with detectable new bone formation (r-axSpA) presented with a longer duration of symptoms and higher CRP levels at baseline. The frequency of A2AP 6Trp carriers tended to be slightly higher in r-axSpA than in nr-axSpA patients (*p* = 0.27).

### 2.2. Serum VEGF Levels in Dependence of A2AP Arg6Trp Genotype

Initially, we analyzed the relation between A2AP Arg6Trp genotype and VEGF elevations and VEGF levels (Table 2).

Overall, the A2AP 6Trp carriage was significantly associated with elevated VEGF levels (cut-off: ≥187 pg/mL) in univariate analyses as well as after adjustment for potentially confounding factors. When considering both axSpA subgroups separately, interestingly, we found that the relation between A2AP Arg6Trp genotype and VEGF elevation was considerably stronger in nr-axSpA patients, i.e., in those patients in whom a significant radiographic bone new formation was still missing.

Subsequently, we used a VEGF cut-off level of 600 pg/mL, which has been described previously to be associated with radiographic spinal progression in axSpA [15]. In this case, no relation between A2AP Arg6Trp genotype and VEGF elevation could be found.

When supplementing possible confounding factors of multivariate analyses by mean intake of non-steroidal anti-inflammatory drugs (NSAIDs) over 2 years, the association strength between A2AP 6Trp carriage and VEGF elevation increased for VEGF ≥ 187 pg/mL (all patients, OR: 2.50, 95% CI: 1.05–5.93; r-axSpA, OR 1.32, 95% CI: 0.42–4.16; nr-axSpA, OR: 5.62, 95% CI: 1.30–24.2) as well as for VEGF ≥ 600 pg/mL (all patients, OR: 2.18, 95% CI: 0.89–5.36; r-axSpA, OR: 1.09, 95% CI: 0.33–3.59; nr-axSpA, OR: 8.70, 95% CI: 1.61–47.1). VEGF levels exhibited no association with mean NSAID (2 years) score (NSAID (2 years) score < 50, VEGF level: 416 ± 327 pg/mL; ≥ 50, VEGF level: 376 ± 232 pg/mL, *p* = 0.97).

Finally, we compared VEGF levels between A2AP 6Trp carriers and A2AP 6Arg/Arg wild types. When considering all axSpA patients, VEGF levels were significantly higher in A2AP 6Trp carriers (455 ± 334 pg/mL) than in A2AP 6Arg/Arg wild types (373 ± 293 pg/mL) (*p* < 0.008). In r-axSpA, there was a statistical trend for higher VEGF levels in A2AP 6Trp carriers (6Trp: 412 ± 251 pg/mL; 6Arg/Arg: 367 ± 253 pg/mL; *p* < 0.08) and in nr-axSpA patients, this difference was more pronounced (6Trp: 524 ± 429 pg/mL; 6Arg/Arg: 379 ± 335 pg/mL; *p* = 0.02).

### 2.3. Serum VEGF Levels in Dependence of Genetic Determinants Related to Fibrinogen Synthesis and Fibrin Clot Formation

Based on data indicating a relation between fibrin and VEGF [11] and the fact that plasmin as well as A2AP exert their effects in dependence on the quality of the fibrin gel matrix [21,24,25,26], we tested the secondary hypothesis that genotypes related to fibrinogen synthesis and/or fibrin clot formation would be associated with VEGF levels.

As given in Table 3, for none of the fibrinogen and factor XIII genotypes tested could we find associations with VEGF elevations. The same holds true when assessing the relation between these genotypes and VEGF levels in two-sample *t*-tests.

We additionally tested the hypothesis that VEGF levels are higher in those individuals potentially predisposed to form loose, fibrinolysis-susceptible fibrin clots using composite genotype constellations consisting of fibrinogen and F13A Val34Leu genotypes, as described previously [23]. None of the composite genotype constellations tested exhibited an association with VEGF levels (data not shown).

### 2.4. Association of VEGF and Radiographic Spinal Progression in axSpA

Elevated VEGF levels have been described to be associated over time with a meaningful increase of heterotopic ossification, so-called spinal progression, in X-rays of axSpA patients, especially in those patients presenting at baseline with syndesmophytes [15].

Therefore, we assessed whether genotypes related to fibrinolysis, fibrinogen synthesis, and/or fibrin clot formation would influence this association between elevated VEGF levels and progression in axSpA (Table 4).

When analyzing the relation between elevated VEGF levels (≥600 pg/mL) with radiographic spinal progression (modified Stoke Ankylosing Spondylitis Spinal Score (mSASSS) ≥ 2 after 2 years) without genotype-stratification, we found a significant association in the total study population (OR: 3.11, 95% CI: 1.02–8.82) and—as has been described previously [15]—this association was extremely more pronounced in those patients presenting at baseline with syndesmophytes (*n* = 41) (OR: 34.7, 95% CI: 3.22–1594). In patients without syndesmophytes at the initial presentation (*n* = 145), an association between VEGF and progression was missing (OR: 0, 95% CI: 0–2.20).

While for most of the genotypes tested, the relation between elevated VEGF levels and radiographic spinal progression was not affected by genotype-based stratification, for FGA rs6050 A>G, a noticeable difference could be found.

In FGA rs6050 AA wild types, elevated VEGF levels were strongly associated with radiographic spinal progression (OR: 5.74, 95% CI: 1.54–20.7), while in carriers of the minor G-allele, this association was not detectable (OR: 0, 95% CI: 0–3.61). The heterogeneity between both FGA rs6050 A>G strata was considerable (test for homogeneity, *p* < 0.04). Given in absolute numbers, when baseline VEGF levels were ≥600 pg/mL, 42.1% (8 of 19) of the axSpA patients with FGA rs6050 AA wild type progressed radiographically after 2 years, while in those axSpA patients carrying at least one FGA rs6050 G-allele, no progress happened (0 of 13). For those patients with lower VEGF levels (<600 pg/mL), in FGA rs6050 AA wild types (11.3%, 9 of 80) and FGA rs6050 G-allele carriers (8.1%, 6 of 74), no relevant difference in progression frequency was found.

For FGA rs2070006 G>A and FGA rs2070016 T>C, the association of VEGF with progression differed between wild types and minor allele carriers as well (Table 4). However, the magnitude of heterogeneity was less pronounced as for FGA rs6050 G>A.

## 3. Discussion

VEGF is central in angiogenesis and tissue repair, and it contributes to inflammatory processes [8]. During wound healing, plasminogen and thrombomodulin are involved in the regulation of VEGF expression, with thrombomodulin presumably mediating plasmin(ogen)-enhanced VEGF expression and wound healing [9]. A lack of A2AP, which is the main inhibitor of plasmin, has been described to be associated with an overexpression of VEGF [2,3]. It is currently unknown whether this phenomenon is related to a reduced inhibition of plasmin, hereby increasing VEGF expression via the aforementioned plasminogen/thrombomodulin signaling or whether A2AP itself directly is involved in VEGF regulation.

After synthesis, A2AP with a N-terminal methionine (Met-A2AP) is secreted, which subsequently is cleaved to Asn-A2AP. Finally, Asn-A2AP is incorporated into the fibrin clot by factor XIII-catalysed crosslinking [16]. The A2AP 6Trp variant (A2AP Arg6Trp, rs2070863) abrogates the initial N-terminal cleavage and therefore results in A2AP-poor, fibrinolysis-susceptible fibrin clots [16]. In our study, we could show that the carriage of A2AP 6Trp is associated with increased VEGF levels in axSpA patients (Table 2). The results of univariate analyses were confirmed in multivariate analysis adjusted for potentially confounding factors (sex, age, CRP, current smoking, NSAID intake), which is of importance, as an influence of A2AP Arg6Trp on inflammatory activity as measured by CRP has been described previously [23]. However, it should be noted that data on comorbidities potentially influencing VEGF levels were not available for analyses. Thus, a potential confounding by comorbidities cannot be excluded. Patients suffering from axSpA are known to frequently present with elevated VEGF levels, and elevated VEGF levels have been described to be associated with an increased risk of disease progression as measured by an increase of spinal bone new formation [15]. The association of the A2AP Arg6Trp genotype with VEGF levels could indicate that fibrin-bound A2AP and the anti-fibrinolytic capacity of the fibrin clot is of importance in VEGF regulation. Alternatively, it could be hypothesized that circulating, completely processed Asn-A2AP is necessary for adequate VEGF-regulation.

As the anti-fibrinolytic capacity of fibrin clots depends on fibrin crosslinking, we subsequently tested whether genotypes known to influence the grade of fibrin crosslinking and the architecture of fibrin networks [19,20,21,27] would influence VEGF levels as well. As given in Table 3, only FGB rs1800790 G>A gave a statistical trend for a reduced frequency of VEGF elevations in A-allele carriers (*p* < 0.07). This genetic variant has been consistently reported to be associated with increased fibrinogen levels [28], and high fibrinogen levels in turn are associated with the formation of fibrin clots with a high density and a high anti-fibrinolytic capacity [20]. Thus, the tendency for lower VEGF levels in FGB rs1800790 A-allele-carrying axSpA patients would be in line with the hypothesis that anti-fibrinolytic capacity is related to VEGF expression.

The heterogeneity in respect of the relation between VEGF elevation (≥600 pg/mL) and radiographic spinal progression (mSASSS ≥ 2 over 2 years) in strata defined by FGA genotypes is a surprising finding, which should be interpreted carefully. These findings originated from exploratory analyses; therefore, the results of testing for heterogeneity (FGA rs6050 A>G, *p* < 0.04; FGA rs2070006 G>A, *p* = 0.05; FGA rs2070016 T>C, *p* < 0.11) should be called noticeable rather than significant. From a clinical viewpoint, it would be of great interest to specify these results in a larger cohort. Until now, the predictive potential of VEGF in respect of radiographic spinal progression in axSpA can be sharpened by adding information on the presence of specific heterotopic ossifications, i.e., the presence of syndesmophytes [15]. If the findings on heterogeneity between carriers and non-carriers of FGA rs6050 G-alleles could be confirmed in larger replication studies, this could help to improve risk stratification and to focus treatment escalation in axSpA patients.

## 4. Materials and Methods

### 4.1. Patients and Clinical Assessment

In this study, we included 186 axSpA patients of the German Spondyloarthritis Inception Cohort (GESPIC), of whom VEGF measurements were possible. Patients were included in GESPIC if they had definite axSpA, either radiographic form (radiographic axSpA, r-axSpA, also referred to as ankylosing spondylitis, AS), or non-radiographic form (non-radiographic axSpA, nr-axSpA). Non-radiographic axSpA is assumed to be an earlier or milder subgroup of axSpA, and patients with nr-axSpA might or might not develop structural bone damage in the axial skeleton later on [13]. Clinical and laboratory data were collected at baseline and every 6 months thereafter, radiographic data (cervical spine lateral view, lumbar spine lateral, and antero-posterior views) were collected at baseline and after 2 years. For the assessment of radiographic spinal progression, i.e., the development of significant structural bone damage, spinal radiographs were collected, digitized, anonymized, and subsequently scored independently by two trained readers in a concealed and randomly selected order according to the modified Stoke Ankylosing Spondylitis Spinal Score (mSASSS) [29]. Meaningful radiographic spinal progression was defined as worsening of the mSASSS score by ≥ 2 points after 2 years. In addition to the mSASSS, we assessed syndesmophytes on both lateral views and lumbar antero-posterior view as previously described [30]. Characterization of the mean intake of non-steroidal anti-inflammatory drugs (NSAIDs) over a period of 2 years as a possible confounding factor was performed as described previously [31].

### 4.2. Quantification of VEGF-A Levels

Serum VEGF-A levels were quantified using an enzyme-linked immunosorbent assay (ELISA) system (R&D Systems, Minneapolis, MN, USA) according to the manufacturer’s instructions.

### 4.3. Genotyping

Fibrinogen (FGA) Thr312Ala (rs6050 A>G), FGA 251 G>A (rs2070006), FGA 3807 T>C (rs2070016), fibrinogen (FGB) -249 C>T (rs1800788), FGB -455 G>A (rs1800790), fibrinogen (FGG) 9340 T>C (rs1049636), factor XIII subunit-A (F13A) Val34Leu (rs5985 G>T), factor XIII B-subunit (F13B) His95Arg (rs6003 A>G), and A2AP Arg6Trp (rs2070863 C>T) genotypes were determined by real-time PCR and melting curve analysis using LightSNiP assays (TIB MOLBIOL GmbH, Berlin, Germany).

### 4.4. Statistical Analyses

Associations of the A2AP Arg6Trp (rs2070863 C>T) genotype as well as the genotypes of the secondary hypotheses with VEGF levels initially were analyzed by Chi^2^-testing using a VEGF cut-off level of 187 pg/mL, according the clinic’s reference range (Charité—Universitätsmedizin Berlin, Berlin, Germany). Subsequently, we used a VEGF cut-off level of 600 pg/mL, which has been used previously for the prediction of radiographic spinal progression in axSpA [15]. Logistic regression analyses on these associations were performed with adjustment for sex, age, current smoking, and C-reactive protein (CRP) levels. For univariate and multivariate analyses, odds ratios (OR) and exact 95% confidence intervals (95% CI) were calculated. Comparisons of VEGF levels between different genotype constellations were performed by two-sample t-tests after log-transformation of VEGF levels.

Additionally, as previously described [23], genotype constellations potentially predisposing for highly crosslinked, dense fibrin structures were defined and used for analyses. These composite genotype constellations were defined as follows: P^FGB rs1800790G>A^ (FGB rs1800790A and F13A 34Val/Val vs. other constellations involving these genotypes), P^FGA rs6050A>G^ (FGA rs6050AA and F13A 34Val/Val vs. other constellations), P^FGB rs1800788C>T^ (FGB rs1800788CC and F13A 34Val/Val vs. other constellations), P^FGA rs2070006G>A^ (FGA rs2070006GG and F13A 34Val/Val vs. other constellations), P^FGA rs2070016T>C^ (FGA rs2070016C and F13A 34Val/Val vs. other constellations).

When evaluating the association of elevated VEGF levels with radiographic spinal progression (mSASSS ≥ 2 after 2 years) by Chi^2^-testing, the homogeneity of genotype-defined strata was evaluated by Breslow–Day testing.

Statistical analyses were performed using Stata statistical software 16 for Macintosh (StataCorp, College Station, TX, USA).

### 4.5. Ethical Approval

The study protocol was approved by the local ethical committee (Charité—Universitätsmedizin Berlin, Berlin, Germany) (188-19 from 2000 and EA1/193/10 from 2012). Written informed consent was provided by all included patients.

## Figures and Tables

**Table 1:** Baseline demographic and clinical characteristics of included patients with axial spondyloarthritis.

Parameter	All Patients (*n* = 186)	r-axSpA (*n* = 105)	nr-axSpA (*n* = 81)
Age, years	37.1 ± 10.5	36.6 ± 11.3	37.8 ± 9.54
Duration of symptoms, years	4.1 ± 2.7	5.0 ± 2.8	3.1 ± 2.1
Male sex, *n* (%)	94 (50.5)	67 (63.8)	27 (33.3)
Smoking, *n* (%)	53 (28.5)	34 (32.4)	19 (23.5)
HLA-B27 carrier, *n* (%)	148 (80.0)	87 (82.9)	61 (76.3)
A2AP 6Trp carrier, *n* (%)	82 (44.1)	50 (47.6)	32 (39.5)
VEGF, pg/mL	409 ± 314	388 ± 252	436 ± 379
CRP, mg/L	9.8 ± 15.7	11.9 ± 16.8	7.0 ± 13.7
mSASSS	4.4 ± 8.7	6.0 ± 10.6	2.3 ± 4.5
Mean NSAID score (2 years)	32.2 ± 27.4	32.2 ± 27.8	32.1 ± 27.0

Continuous variables are presented as mean ± standard deviation. Heterotopic ossification was assessed by modified Stoke Ankylosing Spondylitis Spinal Score (mSASSS). A2AP, α_2_-antiplasmin; CRP, C-reactive protein; mSASSS, modified Stoke Ankylosing Spondylitis Spinal Score; nr-axSpA, non-radiographic axial spondyloarthritis; NSAID, non-steroidal anti-inflammatory drugs; r-axSpA, radiographic axial spondyloarthritis; VEGF, vascular endothelial growth factor.

**Table 2:** Association of A2AP Arg6Trp genotype with VEGF levels.

	A2AP 6Trp Carriage		
	All Patients	r-axSpA	nr-axSpA
	OR (95% CI)	OR (95% CI)	OR (95% CI)
**VEGF ≥ 187 pg/mL**			
univariate	2.50 (1.10–5.95)	1.54 (0.49–5.12)	4.06 (1.13–18.2)
multivariate	2.37 (1.06–5.29)	1.55 (0.52–4.60)	3.56 (1.00–12.6)
**VEGF ≥ 600 pg/mL**			
univariate	1.33 (0.58–3.08)	0.96 (0.27–3.31)	2.00 (0.59–6.84)
multivariate	1.37 (0.62–3.04)	1.00 (0.31–3.04)	2.18 (0.69–6.82)
**VEGF levels [pg/mL]**	Mean ± SD	Mean ± SD	Mean ± SD
total	410 ± 313	388 ± 252	438 ± 377
A2AP 6Arg/Arg	373 ± 293 *	367 ± 253 ^§^	379 ± 335 ^#^
A2AP 6Trp	455 ± 334 *	412 ± 251 ^§^	524 ± 429 ^#^

Analyses on all patients as well as on the subgroup of r-axSpA and nr-axSpA are given. Logistic regression analyses were adjusted for sex, age, CRP and current smoking. 95% CI, 95% confidence interval, A2AP, α_2_-antiplasmin; CRP, C-reactive protein; nr-axSpA, non-radiographic axial spondyloarthritis (SpA); r-axSpA, radiographic spondyloarthritis; SD, standard deviation; VEGF, vascular endothelial growth factor. t-test on log-transformed VEGF levels: * *p* < 0.008 ^§^ *p* < 0.08 ^#^ *p* = 0.02.

**Table 3:** Association of fibrinogen and factor XIII genotypes with VEGF levels.

Genotype	VEGF ≥ 187 pg/mLOR (95% CI)	VEGF ≥ 600 pg/mLOR (95% CI)
FGB rs1800790 G>A	0.52 (0.24–1.12)	0.88 (0.36–2.05)
FGB rs1800788 C>T	1.26 (0.57–2.88)	1.05 (0.43–2.45)
FGA rs6050 A>G	1.10 (0.51–2.36)	0.74 (0.31–1.71)
FGA rs2070006 G>A	0.72 (0.29–1.69)	0.94 (0.39–2.40)
FGA rs2070016 T>C	0.57 (0.26–1.29)	0.83 (0.30–2.10)
FGG rs1049636 T>C	1.00 (0.47–2.17)	0.80 (0.32–1.81)
F13A rs5985 Val34Leu	1.39 (0.64–3.09)	0.52 (0.21–1.25)
F13B rs6003 A>G	1.34 (0.57–3.33)	1.55 (0.63–3.66)

Univariate analyses on all axSpA patients are given in respect of carriage of the respective minor allele. 95% CI, 95% confidence interval, axSpA, axial spondyloarthritis; F13A, factor XIII A-subunit; F13B, factor XIII B-subunit; FGA, fibrinogen; FGB, fibrinogen; FGG, fibrinogen; OR, odds ratio; VEGF, vascular endothelial growth factor.

**Table 4:** Association between VEGF elevation (≥600 pg/mL) and radiographic spinal progression after 2 years in axSpA.

	Radiographic Spinal Progression (mSASSS ≥ 2)	
	Minor Allele	
	Absent OR (95% CI) (*n*)	Present OR (95% CI) (*n*)
A2AP rs2070863 Arg6Trp	2.67 (0.39–13.5) (104)	3.30 (0.70–14.0) (82)
FGB rs1800790 G>A	2.28 (0.45–9.40) (111)	4.67 (0.77–24.7) (74)
FGB rs1800788 C>T	3.07 (0.81–10.6) (118)	3.53 (0.26–34.3)
FGA rs6050 A>G	5.74 * (1.54–20.7) (99)	0 * (0–3.61) (87)
FGA rs2070006 G>A	10.5 ^#^ (1.57–69.9) (56)	1.39 ^#^ (0.23–5.98) (130)
FGA rs2070016 T>C	1.8 ^§^ (0.38–6.87) (134)	10.0 ^§^ (1.25–76.5) (52)
FGG rs1049636 T>C	3.38 (0.62–15.9) (107)	3.22 (0.58–15.2) (79)
F13A rs5985 Val34Leu	4.32 (1.13–15.9) (104)	1.02 (0.02–9.6) (81)
F13B rs6003 A>G	3.03 (0.72–11.1) (131)	3.25 (0.39–22.6) (55)

Univariate analyses on the association between elevated VEGF levels (≥600 pg/mL) and radiographic spinal progression (mSASSS ≥ 2 over 2 years) are given stratified for presence or absence of the respective minor allele.* Test for homogeneity (Breslow–Day): *p* < 0.04 ^#^ Test for homogeneity (Breslow–Day): *p* = 0.05 ^§^ Test for homogeneity (Breslow–Day): *p* < 0.11 95% CI, 95% confidence interval; A2AP, α_2_-antiplasmin; axSpA, axial spondyloarthritis; F13A, factor XIII A-subunit; F13B, factor XIII B-subunit; FGA, fibrinogen; FGB, fibrinogen; FGG, fibrinogen; mSASSS, modified Stoke Ankylosing Spondylitis Spinal Score; OR, odds ratio; VEGF, vascular endothelial growth factor.

## Abbreviations

95% CI	95% confidence interval
A2AP	α_2_-antiplasmin
AS	ankylosing spondylitis
axSpA	axial spondyloarthritis
CRP	C-reactive protein
F13A	factor XIII subunit-A
F13B	factor XIII subunit-B
FGA	α-fibrinogen
FGB	β-fibrinogen
FGG	γ-fibrinogen
GESPIC	German Spondyloarthritis Inception Cohort
mSASSS	modified Stoke Ankylosing Spondylitis Spinal Score
nr-axSpA	non-radiographic axial spondyloarthritis
NSAID	non-steroidal anti-inflammatory drugs
OR	odds ratio
r-axSpA	radiographic axial spondyloarthritis
VEGF	vascular endothelial growth factor
